# Supplementary material for: Carbon footprint comparison of video intubation tools: Disposable laryngoscopes, reusable laryngoscopes, and stylets
Source: PLoS One. 2025 Dec 16;20(12):e0339058. doi: 10.1371/journal.pone.0339058 (PMC12707630; doi:10.1371/journal.pone.0339058)
Supplement: S1 Table — (DOCX) [file pone.0339058.s001.docx]

**S1 Table. Materials of video intubation tools and disposable stylet.**

| **Reusable VL(VL310-3-3)** |  | **Disposable VL(TD-C-IV-3)** |  | **Video stylet (TRS-P2-3)** |  |
| --- | --- | --- | --- | --- | --- |
| **Display component** |  | **Display component** |  | **Display component** |  |
| Polysulfone | 47g | Polysulfone | 47g | Polysulfone | 47g |
| Polycarbonate | 5g | Polycarbonate | 5g | Polycarbonate | 5g |
| LED screen | 20g | LED screen | 20g | LED screen | 20g |
| Lithium polymer battery | 35g | Lithium polymer battery | 35g | Lithium polymer battery | 35g |
| Printed circuit board | 25g | Printed circuit board | 25g | Printed circuit board | 25g |
| Copper | 6g | Copper | 6g | Copper | 6g |
| Silica gel | 3g | Silica gel | 3g | Silica gel | 3g |
|  |  |  |  |  |  |
| **laryngoscope blade components** |  | **laryngoscope blade components** |  | **Stylet components** |  |
| Polyetherimide | 40g | Aluminum alloy | 45g | Polypropylene | 20g |
| Acrylonitrile butadiene styrene resin | 17g | Acrylonitrile butadiene styrene resin | 17g | Polyetherimide | 9g |
| Polysulfone | 1g | Potting glue | 10g | Stainless steel | 30g |
| Copper | 5g | Copper | 5g | Potting glue | 2g |
| Potting glue | 10g | Polysulfone | 1g | Camera lens | 5g |
|  |  | Light-emitting diode | 0.2g |  |  |
|  |  |  |  |  |  |
| **Packaging** |  | **Packaging** |  | **Packaging** |  |
| Corrugated board box | 600g | Corrugated board box | 600g | Corrugated board box | 620g |
| Ethylene Vinyl Acetate cotton | 20g | Ethylene Vinyl Acetate cotton | 20g | Ethylene Vinyl Acetate cotton | 25g |
|  |  |  |  |  |  |
| **Disposable stylet** |  | **Disposable stylet** |  |  |  |
| Aluminum core | 8g | Aluminum core | 8g |  |  |
| Polyvinyl Chloride | 4g | Polyvinyl Chloride | 4g |  |  |
|  |  |  |  |  |  |
|  |  | **Disposable Blade** |  |  |  |
|  |  | Polycarbonate | 11g |  |  |
|  |  | Polyvinyl Chloride | 5g |  |  |
|  |  | Corrugated board box | 210g |  |  |
