# Supplementary material for: Carbon footprint comparison of video intubation tools: Disposable laryngoscopes, reusable laryngoscopes, and stylets
Source: PLoS One. 2025 Dec 16;20(12):e0339058. doi: 10.1371/journal.pone.0339058 (PMC12707630; doi:10.1371/journal.pone.0339058)
Supplement: S2 Table — (DOCX) [file pone.0339058.s002.docx]

**S2 Table. Total Cost of Ownership Comparison Across Video Intubation Devices.**

| **Cost Component​​** | **Video stylet + HLD** | **Video stylet + LTPS** | **Reusable VL + HLD** | **Reusable VL + LTPS** | **Disposable VL** |
| --- | --- | --- | --- | --- | --- |
| **​​Procurement Cost (¥)​​** | 38,000 | 38,000 | 20,000 | 20,000 | 20,000 |
| **​​Per-Use Cost (¥)​​** | 0 | 0 | 4 | 4 | 64 |
| **Sterilization Cost (¥/cycle)​​** | 10 | 30 | 10 | 30 | 0 |
| **​​Waste Disposal Cost (¥/cycle)​​** | 1 | 1 | 5 | 5 | 5 |
| **​​TCO for 500 cycles (¥)​​** | ​​43,500​​ | ​​53,500​​ | ​​29,500​​ | ​​39,500​​ | ​​54,500​​ |
| **​​TCO for 2000 cycles (¥)​​** | ​​60,000​​ | ​​100,000​​ | ​​118,000​​ | ​​158,000​ | 218,000 |

TCO calculation includes procurement, per-use consumables, sterilization, and waste disposal costs over 500 & 2,000 procedure cycles. HLD=high-level disinfection; LTPS=low-temperature plasma sterilization"
