# Supplementary material for: Carbon footprint comparison of video intubation tools: Disposable laryngoscopes, reusable laryngoscopes, and stylets
Source: PLoS One. 2025 Dec 16;20(12):e0339058. doi: 10.1371/journal.pone.0339058 (PMC12707630; doi:10.1371/journal.pone.0339058)
Supplement: S3 Table — (DOCX) [file pone.0339058.s003.docx]

**S3 Table. Empirical Evidence for Device Reuse Lifetime Assumptions​**.

| **Device Model** | **Assigned Lifetime (uses)** | **Evidence Source** | **Evidence Description / Key Parameters** | **Clinical Tracking Data (if applicable)** |
| --- | --- | --- | --- | --- |
| ​**​Reusable Video Laryngoscope (VL310-3-3)​**​ | 2000 | 1. Accelerated lifetime testing 2. Clinical durability tracking (Guangdong Provincial Hospital of Chinese Medicine) | 1. ​**​Accelerated Testing​**​: Simulated mechanical fatigue and electronic component aging; first failure occurred at an equivalent of 2,500 cycles. 2. ​**​Weibull Analysis​**​: Shape parameter β = 2.7, characteristic life η = 2,140 cycles. The lower limit of the 90% confidence interval is ~2,000 cycles. | Prospective observation (2021-2023, n=15 devices). The average retirement life was 2,180 cycles. The primary failure mode was camera module performance degradation (62%). |
| ​**​Reusable Video Stylet (TRS-P2-3)​**​ | 2000 | 1. Accelerated lifetime testing (bending fatigue) 2. Manufacturer's recommendation | 1. ​**​Accelerated Testing​**​: Optical fiber bundle showed signal attenuation after simulated bending for >1800 cycles. 2. ​**​Conservative Adoption​**​: The manufacturer's recommended 2000 cycles was adopted to ensure optical performance. | Data is limited, but in-hospital records indicate an average retirement after 1765 uses, primarily due to optical fiber damage or housing wear. |
| ​**​Single-Use Video Laryngoscope (TD-C-IV-3)​**​ | 2000 | 1. Accelerated lifetime testing 2. Clinical durability tracking (Guangdong Provincial Hospital of Chinese Medicine) | 1. ​**​Accelerated Testing​**​: Simulated mechanical fatigue and electronic component aging; first failure occurred at an equivalent of 2,600 cycles. 2. ​**​Weibull Analysis​**​: Shape parameter β = 2.7, characteristic life η = 2,140 cycles. The lower limit of the 90% confidence interval is ~2,00 cycles. | Prospective observation (2021-2023, n=15 devices). The average retirement life was 2,300 cycles. The primary failure mode was camera module performance degradation (72%). |
