# Supplementary material for: Carbon footprint comparison of video intubation tools: Disposable laryngoscopes, reusable laryngoscopes, and stylets
Source: PLoS One. 2025 Dec 16;20(12):e0339058. doi: 10.1371/journal.pone.0339058 (PMC12707630; doi:10.1371/journal.pone.0339058)
Supplement: S4 Table — (DOCX) [file pone.0339058.s004.docx]

**S4 Table. Life Cycle Inventory Inputs per Cycle for Sterilization Processes.**

| **Parameter** | **LTPS** | **HLD** | **Data Source / Modelling Approach** |
| --- | --- | --- | --- |
| **Electricity (kWh/cycle)​​** | 5.94 kWh​​ | Not Applicable | Custom model, primary data. Based on measured energy consumption per cycle for a STERRAD® 100S system (54-minute cycle at 45°C). |
| **Chemical Consumption​​** | Hydrogen peroxide cartridge, 6 mL | 3% Hydrogen Peroxide (H_2_O_2_), immersion volume: 2.5 L​​ USP-grade sterile water, rinse volume: 5 L​​ | Custom model. Based on equipment technical data sheets. Upstream impacts of chemical manufacturing are modelled using Ecoinvent processes: Hydrogen peroxide, without water, in 50% solution state {GLO}for LTPS, and Glutaraldehyde {GLO}as a proxy for 3% hydrogen peroxide (H_2_O_2_) in HLD. |
| **Rinse Water (L/cycle)​​** | Not Applicable | 5 L (for triple rinsing) | Custom model, primary data. Based on standard protocol for automated reprocessing. |
| **Packaging Material (per device)​​** | Polypropylene/Kraft paper pouch (150 × 200 mm, 0.1 mm thickness) | Polypropylene sterilization pouch (post-sterilization repackaging) | Ecoinvent Process: Modelled using: Packaging film, low density polyethylene {GLO} (as a proxy for polypropylene), mass adjusted for pouch size. |
| **Wastewater Treatment​​** | Not Applicable | ​​Included​​ | Ecoinvent Process: Wastewater, average {Europe without Switzerland} |
| **Chemical Manufacturing Modelling​​** | Not Applicable | ​​Explicitly included​​ | Approach: The environmental burden of producing the consumed 3% H_2_O_2_ solution is included by linking the mass used to the Hydrogen peroxide production pathway in the Ecoinvent database. |

Note: HLD=high-level disinfection; LTPS=low-temperature plasma sterilization
