# Supplementary material for: Carbon footprint comparison of video intubation tools: Disposable laryngoscopes, reusable laryngoscopes, and stylets
Source: PLoS One. 2025 Dec 16;20(12):e0339058. doi: 10.1371/journal.pone.0339058 (PMC12707630; doi:10.1371/journal.pone.0339058)
Supplement: S5 Table — (DOCX) [file pone.0339058.s005.docx]

**S5 Table. Summary of Core LCA Modeling Assumptions​​.**

| **Modeling Element​​** | **​​Assumption/Parameter Setting​​** | **​​Rationale & Data Source​​** |
| --- | --- | --- |
| **​​Functional Unit​​** | One successful tracheal intubation procedure | Standardized clinical output for equitable comparison (ISO 14040:2006) |
| **​​System Boundary​​** | Cradle-to-grave (raw material → disposal) | Comprehensive coverage per ISO 14044:2006  • Material extraction • Manufacturing • Packaging & transport • Use phase (sterilization) • End-of-life |
| **​​Device Lifetimes​​** | •VL310-3-3 Reusable VL: 2,000 cycles, stylets as guide wire: 1use •TRS-P2-3 Reusable stylet: 2,000 cycles • Disposable VL: 2000 cycles, VL blade: 1 use. stylets as guide wire: 1use | Validated via: • Accelerated testing (STERRAD® compatibility) • Clinical tracking at Guangdong Hospital |
| **​​Grid Scenarios​​** | • Base case: China national grid (0.583 kg CO₂e/kWh) • Sensitivity: US/EU/India grids | Regionalization per Ecoinvent 3.8region-specific datasets |
| **Sterilizer Loading​** | Full, 1/2, 1/4, 1/8 load, and single device | Models operational efficiency in clinical practice |

Note: ISO, International Organization for Standardization; VL, video laryngoscope; LCA, life cycle assessment.
