# Supplementary material for: Carbon footprint comparison of video intubation tools: Disposable laryngoscopes, reusable laryngoscopes, and stylets
Source: PLoS One. 2025 Dec 16;20(12):e0339058. doi: 10.1371/journal.pone.0339058 (PMC12707630; doi:10.1371/journal.pone.0339058)
Supplement: S6 Table — (DOCX) [file pone.0339058.s006.docx]

**S6 Table. Percentage Contribution of Life Cycle Stages to Total Carbon Footprint per Device.**

| **Device** | **Life Cycle Stage** | **Contribution (kg CO₂e)** | **​​Contribution (%)​​** | **Hotspot Analysis** |
| --- | --- | --- | --- | --- |
| ​**​Reusable Laryngoscope (VL310-3-3)​**​ | ​**​Material & Manufacturing​**​ | 14.079 | ​**​98.8%​**​ | ​**​Primary Hotspot​**​ |
|  | Transportation | 0.021 | 0.1% | Negligible |
|  | Use (Sterilization) | 0.150 | 1.1% | Secondary for reusables |
|  | End-of-Life | 0.006 | 0.0% | Negligible |
|  | ​**​Total​**​ | ​**​14.256​**​ | ​**​100%​**​ |  |
| ​**​Disposable Laryngoscope (TD-C-IV-3)​**​ | ​**​Material & Manufacturing​**​ | 14.593 | ​**​99.7%​**​ | ​**​Primary Hotspot​**​ |
|  | Transportation | 0.023 | 0.2% | Negligible |
|  | Use (Sterilization) | 0.000 | 0.0% | Not applicable |
|  | End-of-Life | 0.017 | 0.1% | Negligible |
|  | ​**​Total​**​ | ​**​14.633​**​ | ​**​100%​**​ |  |
| ​**​Reusable Stylet (TRS-P2-3)​**​ | ​**​Material & Manufacturing​**​ | 14.315 | ​**​98.8%​**​ | ​**​Primary Hotspot​**​ |
|  | Transportation | 0.021 | 0.1% | Negligible |
|  | Use (Sterilization) | 0.150 | 1.0% | Secondary for reusables |
|  | End-of-Life | 0.003 | 0.0% | Negligible |
|  | ​**​Total​**​ | ​**​14.488​**​ | ​**​100%​**​ |  |

Note:The percentage contribution of each life cycle stage was calculated based on the global warming potential (kg CO₂e) attributable to a single use of the device (i.e., per functional unit). This includes amortizing the impacts of manufacturing and end-of-life for reusable devices over their respective lifetimes.
