# Supplementary material for: Carbon footprint comparison of video intubation tools: Disposable laryngoscopes, reusable laryngoscopes, and stylets
Source: PLoS One. 2025 Dec 16;20(12):e0339058. doi: 10.1371/journal.pone.0339058 (PMC12707630; doi:10.1371/journal.pone.0339058)
Supplement: Attachment — Submitted filename: Comments and Recommendation to the Author plos one.docx [file pone.0339058.s007.docx]

**Comments and Recommendation to the Author:**

**Overall Evaluation:**
This manuscript addresses a timely and relevant issue—the environmental impact of medical devices—using a well-structured life cycle assessment (LCA) framework. The focus on video intubation tools is novel and valuable, particularly in light of growing sustainability concerns in healthcare.

However, a few areas need clarification, refinement, or elaboration to enhance the manuscript's rigor, coherence, and utility for clinical and policy decision-makers.

**Major Comments:**

1. **Scientific Novelty and Relevance (✓ Acceptable)**
   - The study is original and adds value by comparing three video intubation tools using LCA. The inclusion of sensitivity analyses enhances robustness.
   - **Suggestion:** Highlight the clinical implications more directly in the abstract and conclusion (i.e., how should hospitals change purchasing behavior?).
2. **Abstract – Lines 23–51**
   - The abstract is comprehensive but **repetitive** in stating that video stylets are superior.
   - **Revise** to remove redundancy: The sentence beginning *"The video stylet disinfected with hydrogen peroxide..."* and the conclusion at the end repeat similar findings.
3. **Introduction – Lines 55–94**
   - Strong contextualization. However, the transition from background to study rationale could be smoother.
   - **Suggested improvement:** Clarify why environmental comparisons between VL types have not been previously explored in-depth.
   - **Lines 87–94** – Highlight rationale for including all devices from one manufacturer.
4. **Methods – Lines 95–174**
   - The methodology is detailed and adheres to ISO 14040 LCA standards.
   - **Suggestion:** Include a flow diagram of the LCA phases or summarize in a brief table to improve readability for non-expert readers.
   - **Clarify:** How were the assumptions about device lifespans (e.g., 2,000 uses) validated—only through ISO standard or also empirical data?
   - **Lines 111–121** – Emphasize assumptions on device re-use cycles and sterilization scenarios.
5. **Results – Lines 175–243**
   - The results are thorough and include appropriate sensitivity analyses.
   - **Request:** Please add statistical indicators (e.g., confidence intervals) more consistently in Tables and Figures for clarity.
   - **Table 2 & Figure 4** – Suggest clearer presentation with 95% CI and standardized units.
6. **Discussion – Lines 244–298**
   - Well-argued and insightful. The discussion provides meaningful interpretations.
   - **Strengthen:** Consider including economic implications (even a qualitative note), as procurement decisions often balance cost and sustainability.
   - **Lines 259–279** – Highlight design suggestions for future sustainable device development.
7. **Limitations – Lines 285–297**
   - The authors acknowledge key limitations, which is commendable.
   - **Expand briefly** on how future studies could address Scope 3 emissions or the inclusion of battery end-of-life impacts.
8. **Conclusion – Lines 299–305**
   - Clear and supported by data, but somewhat repetitive from earlier sections.
   - **Revise** to add a call-to-action or recommendation for healthcare policy-makers.

**Minor Comments:**

- **Line 84**: Typo — “stylets.” should be “stylets” (extraneous comma).
- **Line 211–214**: Include source(s) for percentage contributions of various lifecycle stages.
- **Line 174**: Add manufacturer name of STERRAD® system for transparency.
