# Supplementary material for: Carbon footprint comparison of video intubation tools: Disposable laryngoscopes, reusable laryngoscopes, and stylets
Source: PLoS One. 2025 Dec 16;20(12):e0339058. doi: 10.1371/journal.pone.0339058 (PMC12707630; doi:10.1371/journal.pone.0339058)
Supplement: Attachment — Submitted filename: Response to reviewers.docx [file pone.0339058.s009.docx]

Dear Editors and Reviewrs:

We sincerely appreciate the opportunity you have provided us to revise our manuscript.

We would like to submit an accompanying manuscript titled "***Carbon Footprint Comparison of Video Intubation Tools: Disposable Laryngoscopes, Reusable Laryngoscopes, and Stylets***" (ID: PONE-D-25-19315). According to the reviewers' comments, we have made the most effort to revise the manuscript in order to satisfy the demand of ***PLOS ONE***. Our specific responses are outlined below. The modifications of the text in the revised version are highlighted in red and the references in yellow.

All authors have approved the revised version and consented to its submission. There are no conflicts of interest to declare.

Thank you for your thoughtful consideration. We eagerly anticipate your correspondence.

July 21, 2025（2025-07-21）

Authors: Danyang Pan; Yating Yang; Sirui Chen; Jinhe Deng; Gaofeng Zhao; Min Zhong

Corresponding author: Gaofeng Zhao

E-mail: zhaogaofengszy@163.com

Sincerely

Gaofeng Zhao

Dear managing editor

We have studied the valuable comments from you carefully, and tried our best to revise the manuscript. The point to point response to you are listed as following:

**Editor:**

**Comment 1:** Please ensure that your manuscript meets PLOS ONE's style requirements, including those for file naming. The PLOS ONE style templates can be found at https://journals.plos.org/plosone/s/file?id=wjVg/PLOSOne_formatting_sample_main_body.pdf & <https://journals.plos.org/plosone/s/file?id=ba62/PLOSOne_formatting_sample_title_authors_affiliations.pdf.>

**Response 1:**

We sincerely appreciate the meticulous style check. The manuscript has been rigorously reformatted to comply with PLOS ONE style requirements.

**Comment 2:** In the online submission form, you indicated that your data is available only on request from a third party. Please note that your Data Availability Statement is currently missing the name of the contact details for the third party, such as an email address or a link to where data requests can be made. Please update your statement with the missing information.

**Response 2:**

We appreciate this important clarification. All data underlying this study are publicly accessible through the following repositorie. The Data Availability Statement has been updated as follows in the manuscript and submission system: all data used in this study are publicly available. Carbon emission data were primarily derived from the Ecoinvent 3.8 database; researchers seeking reproducibility of this database must obtain it directly from Simapro at https://simapro.com/contact/. The processed analysis datasets (laryngoscope manufacturing raw data, price data) are provided as Supporting Information Files S1 and S2, and all raw data are available on Figshare at https://figshare.com/articles/dataset/29816855 [doi:10.6084/m9.figshare.29816855].

**Comment 3:** PLOS requires an ORCID iD for the corresponding author in Editorial Manager on papers submitted after December 6th, 2016. Please ensure that you have an ORCID iD and that it is validated in Editorial Manager. To do this, go to ‘Update my Information’ (in the upper left-hand corner of the main menu), and click on the Fetch/Validate link next to the ORCID field. This will take you to the ORCID site and allow you to create a new iD or authenticate a pre-existing iD in Editorial Manager.

**Response 3:**

We confirm that the corresponding author's ORCID iD (0009-0009-1875-586X) has been successfully validated. Last authenticated: 2025-07-21

**Comment 4:** Please include captions for your Supporting Information files at the end of your manuscript, and update any in-text citations to match accordingly. Please see our Supporting Information guidelines: http://journals.plos.org/plosone/s/supporting-information.

**Response 4:**

We have meticulously addressed the supporting information requirements through the following revisions, and added "Supporting Information" immediately after references (Page 26, line 503):

(1) Table Renaming & Relocation

Original Table 1 has been moved to supporting materials and renamed as:
S1_Table. Materials of video intubation tools and disposable stylet

(2) New Supplementary Table:

Added S2_Table. Total Cost of Ownership Comparison Across Video Intubation Devices.

**Comment 5:** Please review your reference list to ensure that it is complete and correct. If you have cited papers that have been retracted, please include the rationale for doing so in the manuscript text, or remove these references and replace them with relevant current references. Any changes to the reference list should be mentioned in the rebuttal letter that accompanies your revised manuscript. If you need to cite a retracted article, indicate the article’s retracted status in the References list and also include a citation and full reference for the retraction notice.

**Response 5:**

We thank the editor for this critical quality control reminder. The reference list has been rigorously re-examined through a three-step verification protocol:

(1) Cross-checking all references against PubMed databases
(2) Confirming publication status via journal official websites

All modifications to the reference list have been documented in the subsequent responses to editorial comments within the rebuttal letter.

**Reviewer #1**

This manuscript addresses a timely and relevant issue—the environmental impact of medical devices—using a well-structured life cycle assessment (LCA) framework. The focus on video intubation tools is novel and valuable, particularly in light of growing sustainability concerns in healthcare.

However, a few areas need clarification, refinement, or elaboration to enhance the manuscript's rigor, coherence, and utility for clinical and policy decision-makers.

**Major Comments:**

**Comment 1:**

1. Scientific Novelty and Relevance (✓ Acceptable)

- The study is original and adds value by comparing three video intubation tools using LCA. The inclusion of sensitivity analyses enhances robustness.
- Suggestion: Highlight the clinical implications more directly in the abstract and conclusion (i.e., how should hospitals change purchasing behavior?).

**Response 1:**

We thank the reviewers for acknowledging the study's novelty and technical rigor. We have made the necessary adjustments to the abstract and conclusion to fulfill the above-mentioned requirements. (Page 3, line 51-56 ; Page 19, line 349-358).

**Comment 2:**

2. Abstract – Lines 23–51

o The abstract is comprehensive but repetitive in stating that video stylets are superior.

o Revise to remove redundancy: The sentence beginning "The video stylet disinfected with hydrogen peroxide..." and the conclusion at the end repeat similar findings.

**Response 2:**

Regarding the duplication concern in the Abstract we have implemented comprehensive revisions in coordination with the prior editorial suggestion (Page 3, line 51-56).

**Comment 3:**

3. Introduction – Lines 55–94

o Strong contextualization. However, the transition from background to study rationale could be smoother.

o Suggested improvement: Clarify why environmental comparisons between VL types have not been previously explored in-depth.

o Lines 87–94 – Highlight rationale for including all devices from one manufacturer.

**Response 3:**

We thank the reviewer for this constructive suggestion. The Introduction section (Lines 55-94) has been revised to enhance the rationale transition. (Page 5, line 92-97).

The manufacturer selection rationale has been strengthened to explicitly align with **ISO 14044** requirements for comparative life cycle assessment of functionally equivalent products. (Page 5, line 101-106).

**Comment 4:**

4. 1 The methodology is detailed and adheres to ISO 14040 LCA standards.

Suggestion: Include a flow diagram of the LCA phases or summarize in a brief table to improve readability for non-expert readers.

**Response4.1:**

We sincerely appreciate this constructive suggestion. As requested, we have incorporated Figure 3: Integrated Life Cycle Assessment Framework for Video Intubation Tools in the revised manuscript ((Page 7 line 146-147). This diagram visually synthesizes the complete LCA workflow.

Consequential figure renumbering has been implemented throughout:

- Original Figure 1 → Revised Figure 2
- Original Figure 2 → Revised Figure 1
- Original Figure 3 → Revised Figure 4
- Original Figure 4 → Revised Figure 5

4.2 Clarify: How were the assumptions about device lifespans (e.g., 2,000 uses) validated—only through ISO standard or also empirical data?

**Response 4.2:**

We express our appreciation for the reviewer's methodological inquiry regarding the device lifespan parameter. This parameter (2,000 uses) was validated through a hybrid methodology incorporating both Accelerated Mechanical Testing and Clinical Durability Tracking. The mechanical testing data were derived from manufacturer validation dossiers (Zhejiang UE Medical, 2023-Q4), evaluating critical electronic components (CMOS sensors, battery connectors) and mechanical stress thresholds under simulated intubation cycles; proprietary confidentiality restrictions prevent full protocol disclosure. Complementarily, clinical durability tracking at Guangdong Provincial Hospital of Chinese Medicine - a tertiary referral center (2021-2023). Evidence-weighted integration of these datasets through Weibull survival analysis (β=2.7, η=2140) confirmed the conservative selection of 2,000 operational cycles, representing the lower 90% confidence interval of clinical performance data.

**Comment 5:**

5. Results – Lines 175–243

o The results are thorough and include appropriate sensitivity analyses.

o Request: Please add statistical indicators (e.g., confidence intervals) more consistently in Tables and Figures for clarity.

o Table 2 & Figure 4 – Suggest clearer presentation with 95% CI and standardized units.

**Response 5:**

We sincerely appreciate this constructive suggestion for enhanced statistical rigor. The following revisions have been implemented per the reviewer's guidance:

- Table 1: Supplemented all values with 95% confidence intervals ( Original Table 2 → Revised  Table 1).
- Figure 5: Column charts have been updated with error bars denoting 95% CIs ( Original Figure 4 → Revised Figure 5)

**Comment 6:**

6. Discussion – Lines 244–298

Well-argued and insightful. The discussion provides meaningful interpretations.

o Strengthen: Consider including economic implications (even a qualitative note), as procurement decisions often balance cost and sustainability.

o Lines 259–279 – Highlight design suggestions for future sustainable device development.

**Response 6:**

We thank the reviewer for this valuable suggestion. The following enhancements addressing economic implications have been incorporated:

1. A Total Cost of Ownership (TCO) comparison table evaluating video intubation devices across procurement, consumables, sterilization, and waste management expenditures;（S1 **Table 2: Total Cost of Ownership Comparison Across Video Intubation Device**）

2. Qualitative economic impact assessments in both Results (Page 15, line 256-273).and Discussion (Page 18, line 341-348).

**Comment 7:**

7. Limitations – Lines 285–297

o The authors acknowledge key limitations, which is commendable.

o Expand briefly on how future studies could address Scope 3 emissions or the inclusion of battery end-of-life impacts.

**Response 7:**

We have expanded and supplemented the manuscript about Scope 3 emissions or the inclusion of battery end-of-life impacts for future research addressing (Page 17, line 321-327).

**Comment 8:**

8. Conclusion – Lines 299–305

o Clear and supported by data, but somewhat repetitive from earlier sections.

o Revise to add a call-to-action or recommendation for healthcare policy-makers.

**Response 8:**

We sincerely appreciate the reviewer's insightful guidance, which has significantly strengthened our manuscript. In direct response to your recommendations:

1. Redundant passages duplicating prior content have been carefully removed to enhance narrative concision and reader focus.
2. Healthcare policy-formulation guidance with actionable recommendations for regulatory implementation has been incorporated. (Page 19, line 349).

**Comment 9:**

**Minor Comments:**

• Line 84: Typo — “stylets.” should be “stylets” (extraneous comma).

**Response 9.1:**

During our diligent verification of the initial manuscript, no extraneous comma was identified at Line 84. However, a superfluous comma at Line 88 (' stylets.,') has been proactively corrected through its deletion. (Page 5, line 100).

• Line 211–214: Include source(s) for percentage contributions of various lifecycle stages.

**Response 9.2:**

We quantified the carbon footprint distribution across life cycle stages through ISO 14044, yielding precise percentage contributions of emissions for each life cycle phase across all evaluated laryngoscope device types.

• Line 174: Add manufacturer name of STERRAD® system for transparency.

**Response 9.3:**

The manufacturer identification for the STERRAD® sterilization system has been explicitly added as:"Advanced Sterilization Products , Division of Johnson & Johnson". (Page 9, line 190).

**Reviewer: 2**

I read with interest the manuscript by Zhao et all which compares the carbon footprint of 3 videointubation tools. The topic is important and timely. The manuscript is systematic in its approach and well written. I have a few questions/suggestions regarding the manuscript.

**Comments 1:**

1. Abstract: please define ISO 14040

**Response 1:**

We have supplemented the manuscript with a formal definition of ISO 14040, explicitly articulating its framework as the internationally standardized methodology for conducting life cycle assessments across four iterative phases：(1) Goal and scope definition, (2) Life cycle inventory analysis (LCI), (3) Life cycle impact assessment (LCIA), and (4) Interpretation. (Page 2, line 29)

**Comments 2:**

2. Please elaborate on the meaning of “prevalent” video intubation tools. Are use of these particular VL tools prevalent in China alone? Worldwide? What % of intubations are performed with these devices?

**Response 2:**

We thank the reviewer for this critical request for epidemiological context. The term "prevalent video intubation tools" refers to device categories demonstrating >50% adoption rates across global clinical practice, with specific validation as follows:

Internationally, video laryngoscopes are classified into three structural categories based on technical configurations:

(1) Traditional Laryngoscope Add-ons (e.g., C-MAC® and McGrath™ MAC), which retrofit miniature cameras onto Macintosh blades, preserving conventional intubation techniques but requiring external displays;
(2) Integrated Video Laryngoscopes (e.g., GlideScope® and KingVision®), featuring combined camera, light source, and display within a single handle for high portability (≤300g), with subtypes utilizing either reusable polymer blades (e.g., UE Vision) or single-use blades (e.g., McGrath™ MAC Single Use);
(3) Video Stylets (e.g., Levitan™ Scope and UE Video Stylet), employing flexible video-equipped stylets for difficult airway management without conventional blade structures.

Due to portability limitations, add-on devices have declined globally. This study quantifies the carbon footprints of three clinically dominant types in China and internationally: integrated VLs with single-use blades, integrated VLs with reusable blades, and video stylets. UE Medical products were selected for controlled comparison as they represent >70% of China's laryngoscope market (MedTech Analytics 2023) and align with ISO 14044:2006 requirements for homogeneity in upstream supply chains and core modules when assessing functionally equivalent medical devices.

**Comments 3:**

3. Half load sterilization should be defined in the abstract/text. Does this refer to batch sterilization?

**Response 3:**

We thank the reviewer for requesting clarification on sterilization terminology. The term **"half-load sterilization"** has been formally defin**ed as "50% chamber utilization"** **i**n abstract. (Page 2, line 44) The term "half-load sterilization" refers specifically to batch sterilization efficiency, defined as the ratio of actual device volume to rated chamber capacity within low-temperature plasma sterilizers. This metric quantifies operational resource utilization, where a 50% load indicates half the sterilizer's maximum device capacity is utilized per cycle. For instance, processing 40 laryngoscopes in a sterilizer chamber rated for 80 devices constitutes a 50% load scenario.

**Comments 4:**

4. Page 6: The statistical analyses used for comparison should be detailed in the manuscript. Merely mentioning that SimaPro and Ecoinvent were used is insufficient. Furthermore this software is likely to be unfamiliar to many readers

**Response 4:**

We gratefully acknowledge this constructive suggestion. Per the reviewer's guidance, we have:

- Supplemented detailed introductions to SimaPro (v9.4.0) and Ecoinvent (v3.8) in Methods; (Page 7, line 143-146).
- Developed an Integrated Life Cycle Assessment Framework for Video Intubation Tools (Fig. 3) to facilitate sreader comprehension of the workflow.

**Comments 5:**

5. Page 9, line 146: Please provide a reference for the equivalent first pass success rate of the 3 tools being compared.

**Response 5:**

We thank the reviewer for highlighting this essential clinical validation requirement. The following evidence supporting equivalent first-pass success rates across the three device categories has been incorporated:

(1) For video laryngoscopes (McGrath™ MAC, AIRWAY SCOPE®, AceScope®)

"Randomized crossover simulation studies confirm comparable first-pass success rates among mainstream video laryngoscopes (97-99%) without statistically significant differences (p>0.05) in novice operators."
Reference:
Yahagi M, et al. BMC Anesthesiol 2025;25:212. doi:10.1186/s12871-025-03094-y
(Added to Line xxx and Reference #24)

(2) For video stylets vs. video laryngoscopes in difficult airways

"Meta-analysis of cervical immobilization trials demonstrates equivalent first-attempt success rates between video stylets and video laryngoscopes (RR=0.96, 95% CI: 0.90–1.03, p=0.3)."
Reference:
Lee A, et al. Anaesth Crit Care Pain Med 2021;10:100965. doi:10.1016/j.accpm.2021.100965
(Added to Linxxx and Reference #25)

**Comments 6:**

6. Page15, line 255: Have other studies examined/compared the carbon footprint of videointubation tools? If yes then the current work should be compared/contrasted with the prior work in the discussion. If not then the novelty of this work should be emphasized.

**Response 6:**

We thank the reviewer for this critical contextualization request. Through a systematic literature review spanning PubMed, Web of Science, and Ecoinvent literature databases (2000-2024) using keywords *"carbon footprint" AND ("video laryngoscopy" OR "video stylet") AND "life cycle assessment"*, we confirmed that **no prior studies have conducted comparative LCAs across the three dominant video intubation modalities** (reusable VLs, single-use VLs, video stylets). We have revised the introduction sections to highlight the study's distinctive scholarly novelty (Page 5, line 94).

**Comments 7:**

7. Can the findings be generalized to other video intubation tools/devices? What are the practical implications of this study?

**Response 7:**

Our study demonstrates the ecological and economic benefit of using reusable video stylets reprocessed via hydrogen peroxide high-level disinfection (HLD). These results may vary depending on the region, country and institution according to various parameters, e.g., negotiated prices and type of energy used. Life cycle assessment (LCA) and life cycle costing (LCC) establish methodologically sound frameworks for evaluating environmental-economic tradeoffs in medical device procurement. Given institutional operational heterogeneity, this work systematically quantified: i) sterilization modalities (LTPS/HLD), ii) LTPS loading efficiency gradients (0–100% capacity), and iii) region-specific electricity grids (China / India / European/ United States) via Monte Carlo sensitivity analysis—increasing cross-institutional generalizability of findings.

Our study suggests potential practical implications through three exploratory pathways:

(1) A clinically applicable decision framework for sustainable device selection, potentially shifting procurement practices toward integrated eco-clinical evaluations in selected settings.
(2) Results may inform beyond binary choices by highlighting significant carbon contributors in product life cycles. Manufacturing appears dominant for single-use devices (observed 73-82% for SUD-L in this analysis), aligning with prior LCAs of disposable medical products including laryngoscopes, LMAs, surgical drapes, and isolation gowns.
(3) Pinpointing carbon hotspots facilitates defining targeted research priorities for decarbonizing video laryngoscopes across their life cycles.

**Comments 8:**

8. What are suggested next steps in research based on the findings of this study?

**Response 8:**

We sincerely appreciate the reviewer's insightful suggestions for advancing this field. Building upon our core finding, we propose the following research directions:

(1) Material-Specific Carbon Impact Analysis Quantify the carbon footprint differentials across video stylet material compositions (e.g., nitinol vs. tranditional cores) through cradle-to-grave LCA, targeting >15% emission reduction via sustainable material substitution. (page 16, line 295)
(2) Eco-Efficient Sterilization Technology Assessment Compare novel low-temperature sterilization technologies (e.g., ozone, UV-C pulsed light) against conventional HLD/LTPS systems, evaluating both microbial efficacy and carbon abatement potential. (page 17, line 307-312)
(3) Carbon-Footprint-Stratified Procurement Index Development Establish a clinically validated Green Procurement Index (GPI) incorporating: Carbon intensity tiers (kg CO₂e/procedure) Recyclability metrics (material recovery rate %) Operational efficiency scores (procedures/hour)，and price. (page 19, line 354-357).
(4) Operational Workflow Optimization Studies Model the carbon reduction potential of surgical turnover acceleration.(page 17, line 320-322).

We hope that these revisions address the reviewer’s concerns and enhance the completeness and academic value of the study.

Thank you once again for your valuable suggestions!
