# Supplementary material for: Carbon footprint comparison of video intubation tools: Disposable laryngoscopes, reusable laryngoscopes, and stylets
Source: PLoS One. 2025 Dec 16;20(12):e0339058. doi: 10.1371/journal.pone.0339058 (PMC12707630; doi:10.1371/journal.pone.0339058)
Supplement: Attachment — Submitted filename: Response to reviewers(V2).docx [file pone.0339058.s010.docx]

Dear Editors and Reviewrs:

We sincerely appreciate the opportunity you have provided us to revise our manuscript.

We would like to submit an accompanying manuscript titled "***Carbon Footprint Comparison of Video Intubation Tools: Disposable Laryngoscopes, Reusable Laryngoscopes, and Stylets***" (ID: PONE-D-25-19315R1). According to the reviewers' comments, we have made the most effort to revise the manuscript in order to satisfy the demand of ***PLOS ONE***. Our specific responses are outlined below. The modifications of the text in the revised version are highlighted in blue.

All authors have approved the revised version and consented to its submission. There are no conflicts of interest to declare.

Thank you for your thoughtful consideration. We eagerly anticipate your correspondence.

November 11, 2025（2025-11-11）

Authors: Danyang Pan; Yating Yang; Sirui Chen; Jinhe Deng; Gaofeng Zhao; Min Zhong

Corresponding author: Gaofeng Zhao

E-mail: zhaogaofengszy@163.com

Sincerely

Gaofeng Zhao

**Reviewer #3**

*** Major issues to address (required before acceptance)**

**Comment 1:**

1. Inventory transparency and reproducibility

- Provide the processed SimaPro input tables or a plain spreadsheet listing every inventory input used in the primary scenarios (component masses, material types, transport distances, per‑cycle energy and chemical consumptions, sterilizer loading assumptions, reuse counts). If manufacturer raw dossiers are proprietary, supply aggregated redacted inputs sufficient to reproduce the reported CO2e numbers.

- Deposit these files in the Figshare DOI already cited or another public repository and add accession links in the Data Availability Statement.

**Response 1:**

We thank the reviewer for this critical suggestion to enhance the transparency and reproducibility of our life cycle assessment. We have taken the following actions to address the point:

1. Creation of Detailed Inventory Tables: As suggested, we have created two comprehensive data files:

1.1 Primary_LCI_Data.xlsx: This spreadsheet lists every inventory input for the primary scenarios, including component masses, material types, transport distances, per-cycle energy and chemical consumptions for reprocessing, sterilizer loading assumptions, and reuse counts.

1.2 S1 Table. Materials of video intubation tools and disposable stylet.docx: For any parameters derived from manufacturer dossiers that are proprietary, we have provided aggregated and redacted values that maintain commercial confidentiality while being fully sufficient to reproduce the CO2e results reported in the manuscript.

2. Deposition in Public Repository: We have deposited the file (Primary_LCI_Data.xlsx) into the existing Figshare repository (DOI: 10.6084/m9.figshare.29816855).
3. Update to the Manuscript: We have updated the Data Availability Statement in the manuscript to explicitly mention the availability of these new LCI files and their direct link to reproducing the study's findings.

**Comment 2:**

2. Device lifetime evidence and sensitivity

- Report a short table in the Supplementary Information summarizing the empirical evidence used to set reuse lifetimes (accelerated testing summary, Weibull parameters or summary statistics, clinical durability tracking numbers).

- Add a sensitivity showing how the primary rankings change if lifetimes differ by ±25–50%.

**Response 2:**

We sincerely thank the reviewer for this valuable suggestion to strengthen the robustness of our findings. We have fully addressed both points as follows:

1. Empirical Evidence for Device Lifetime:

We have created a new Supplementary Table S3 titled “Empirical evidence summary for setting device reuse lifetimes”. This table concisely summarizes the evidence base for each device’s lifetime, including:

∙Key parameters from accelerated testing (e.g., simulated cycles to failure).

∙Weibull parameters (shape parameter β, characteristic life η) and confidence intervals derived from clinical durability tracking data from Guangdong Provincial Hospital of Chinese Medicine.

∙A description of the clinical tracking program itself.

2. Sensitivity Analysis on Lifetime Assumptions:

As suggested, we have performed a comprehensive sensitivity analysis by varying the lifetimes of reusable devices by ±25% and ±50%. The key results of this analysis have been incorporated directly into Table 1 of the main manuscript. Furthermore, these findings are discussed in the Results section where we confirm that the primary conclusions of the study remain unchanged across all tested scenarios, thus demonstrating their robustness.

We have also updated the Methods section to describe the approach for the sensitivity analysis.

**Comment 3:**

3. Sterilization modelling detail

- Provide a line-item account for LTPS and HLD: per-cycle energy (kWh), chemical mass/volume, rinse water, packaging per cycle, and whether those flows were taken from Ecoinvent processes or modelled custom.

- State clearly how chemical manufacture and wastewater treatment impacts were modelled. Add these inputs to the SI inventory spreadsheet.

**Response 3:**

We thank the reviewer for highlighting the need for greater transparency in our sterilization modelling. We have thoroughly addressed this comment by providing a line-item account of the inputs and clarifying the modelling approach as follows:

1. Created a new Supplementary Table S4 titled "Life Cycle Inventory Inputs per Cycle for Sterilization Processes," which provides a comprehensive, line-item breakdown of all parameters for both LTPS and HLD, including energy consumption, chemical volumes, rinse water, and packaging, with clear designation of data sources (Ecoinvent processes or custom data).
2. Added a dedicated methodological statement in the main text (Methods section) that explicitly directs readers to this table.

**Comment 4:**

4. Scope 3 and battery end-of-life bounding analysis

Include at least one bounding sensitivity that adds plausible Scope 3 manufacturing or battery EoL burdens (e.g., manufacturing +10% and +25%, and a battery-EoL scenario). Report whether the main conclusion (stylet + HLD lowest CO2e under high throughput) still holds under those bounds.

**Response4:**

We thank the reviewer for this critical suggestion to test the robustness of our conclusions. We have fully addressed the comment as follows:

1. Scope 3 Manufacturing Sensitivity Analysis: As suggested, we conducted a bounding analysis by increasing the manufacturing-phase (Scope 3) carbon footprint of all electronic components by +10% and +25%. The results confirm that the relative ranking of the devices remains unchanged, and our primary conclusion—that the video stylet with HLD has the lowest carbon footprint under high-throughput conditions—is robust under these expanded Scope 3 boundaries. The key results of this analysis have been incorporated directly into Table 1 of the main manuscript.

2. Battery End-of-Life (EoL) Scenario Assessment: In response to the reviewer's point, we also performed a detailed assessment of the battery EoL. We modeled the recycling of the lithium-ion battery (mass: 35.7 g) using two distinct processes available in the Ecoinvent database. The resulting carbon footprint for both recycling scenarios was on the order of 10⁻⁶ kg CO₂e per functional unit, which is negligible (less than 0.001%) compared to the total footprint of the devices (which are on the order of 10⁰-10¹ kg CO₂e).

Given that the battery mass is identical across all video-enabled devices and its EoL impact is vanishingly small, the inclusion of this EoL phase does not alter the comparative results in any meaningful way. Therefore, in the interest of clarity and focus, we have not added this specific scenario to the main sensitivity analysis in the manuscript, as it does not challenge the robustness of our conclusions. We sincerely hope this explanation, supported by our modeling, addresses the reviewer's concern regarding the battery EoL.

we are grateful for the opportunity to clarify our approach to the battery EoL.

**Comment 5:**

5. Resolve numeric inconsistencies and formatting errors

- Fix obvious typos (for example “18'1.45” → “181.45”), standardize confidence interval formatting across tables/figures, and ensure figure/table numbering and captions match the text. Ensure all units and functional‑unit statements are consistent.

**Response 5:**

We sincerely thank the reviewer for their meticulous attention to detail. We have conducted a comprehensive, line-by-line review of the manuscript to address all typographical and formatting issues. The corrections made are summarized below:

1. Specific Typo Correction: The value “18'1.45”has been corrected to “181.45”in the results section and corresponding table.

2. Global Formatting Standardization:

Confidence Intervals: All confidence intervals across the text, tables, and figures have been standardized to the format “value (95% CI, lower to upper)”(e.g., 27.32 (95% CI, 26.6 to 28.1)).

∙ Units: All units have been verified for consistency, ensuring the use of “kg CO₂e”, “kWh”, and “L”throughout the manuscript.

∙ Functional Unit: The functional unit declaration (per procedure) has been consistently reiterated in the abstract, methods, and captions of all relevant figures and tables.

3. Cross-Referencing Check: We have verified that all in-text citations of figures and tables (e.g., Figure 3, Table 1) correctly match their assigned numbers and captions. The numbering sequence for all figures, tables, and supplementary materials has been confirmed to be continuous and correct.

**Comment 6:**

**6. * Minor Suggestions (recommended)**

6.1 Add a concise Methods table summarizing modelling assumptions (functional unit, system boundary, device lifetimes, grid cases, sterilizer loading cases).

6.2 Present a one‑page reconciliation (bar chart or table) showing percentage contributions (material, manufacturing, sterilization, transport, EoL) for each device to highlight hotspots at a glance.

6.3 Confirm and state which ISO procedures (ISO 14040 / 14044) were followed and where interpretation choices were made.

6.4 Include a completed LCA reporting checklist (or STROBE if applicable) in the SI.

6.5 Consider a short paragraph in Discussion about generalizability given single‑manufacturer sourcing and how procurement decisions might adapt the framework to other suppliers.

**Response 6:**

We thank the reviewer for these insightful recommendations, which have significantly enhanced the clarity and rigor of our manuscript. We have addressed each point as follows:

6.1. Concise Methods Table

we have created a new Supplementary Table S4 (entitled "Summary of Core LCA Modeling Assumptions") and inserted it into the Methods section. This table concisely summarizes all the key parameters requested by the reviewer: functional unit, system boundary, device lifetimes, grid electricity scenarios, sterilizer loading cases.

6.2. Hotspot Analysis Chart

We thank the reviewer for this valuable suggestion to enhance the clarity of our environmental impact analysis. In accordance with your recommendation, we have added a new Supplementary Table S6 entitled "Percentage Contribution of Life Cycle Stages to Total Carbon Footprint per Device."

This table provides a clear, quantifiable breakdown of the relative contributions of each life cycle stage (material production, manufacturing, sterilization, transport, and end-of-life) to the total carbon footprint of each device configuration. The tabular format allows for immediate identification of environmental hotspots, confirming that material production and manufacturing phases constitute the dominant contributions across all device types.

We have referenced this table in the Results section where we discuss the carbon footprint analysis, and it serves as the basis for our hotspot identification in the Discussion. We believe this addition successfully addresses your request for an at-a-glance overview of the contribution analysis.

6.3. ISO Procedure Statement

We thank the reviewer for raising this critical point regarding methodological rigor and transparency. We are pleased to confirm that our study was conducted in full accordance with the principles and framework of the international standards ISO 14040:2006 and ISO 14044:2006.

To enhance clarity, we have now added an explicit statement of compliance in the Methods section (under the "Life Cycle Assessment" subsection):

"This life cycle assessment was conducted in accordance with the principles, framework, and requirements of the international standards ISO 14040:2006 and ISO 14044:2006."

Furthermore, we have meticulously documented the key interpretation choices made during the study, as guided by the standards. These are now detailed in the manuscript as follows:

Goal and Scope Definition:

Functional Unit: The functional unit was defined as 'one successful tracheal intubation procedure'. This choice ensures comparisons are based on equivalent clinical output, a cornerstone of comparative LCA (ISO 14040:2006, §4.2.2).

System Boundary: A 'cradle-to-grave' boundary was selected, encompassing raw material extraction, manufacturing, transportation, use phase (including sterilization), and end-of-life disposal. This provides a comprehensive assessment as recommended by the standards (ISO 14044:2006, §4.2.3.3).

Life Cycle Inventory (LCI) & Impact Assessment (LCIA):

Allocation: For reusable devices, environmental burdens from manufacturing and end-of-life were allocated per use based on the device's validated lifetime (e.g., 2000 uses). This approach aligns with the standard's hierarchy, prioritizing physical relationships (ISO 14044:2006, §4.3.4.2).

Data Selection: When primary data from manufacturers were unavailable, secondary data from the Ecoinvent 3.8 database were used. This is a standard practice to ensure feasibility, and we have clearly indicated the use of such data in the manuscript and supplementary materials (ISO 14044:2006, §4.3.3.3).

Impact Category: The analysis focused on global warming potential (GWP) in kg CO₂e. This selection is justified as it is the most relevant and decision-useful category for carbon footprint studies and climate policy.

Interpretation:

Sensitivity and Uncertainty: As mandated by the standards (ISO 14044:2006, §4.5), we conducted extensive sensitivity analyses on critical parameters (device lifetime, grid electricity, sterilizer loading) to test the robustness of our conclusions and to assess the influence of these interpretation choices.

We believe these additions significantly enhance the transparency and reproducibility of our work, and we are grateful for the reviewer's guidance in helping us make this explicit.

6.4. LCA Reporting Checklist

We have completed a comprehensive LCA reporting checklist and included it as Supplementary File 1 (titled "Completed LCA Reporting Checklist").

The checklist we utilized is adapted from the consensus reporting guidelines for Life Cycle Assessment, which aligns with the ISO 14040/14044 standards. This checklist ensures that all critical methodological aspects of our study—including goal and scope definition, life cycle inventory, impact assessment, and interpretation—are thoroughly and transparently documented.

To ensure comprehensive methodological reporting, a direct reference to this checklist has been incorporated into the Methods section.

6.5. Discussion on Generalizability

We sincerely thank the reviewer for this insightful suggestion, which has helped us significantly elevate the discussion of our work's broader impact. We have fully addressed this point by adding a new paragraph to the Discussion section under the subheading "Generalizability and Implications for Sustainable Procurement."

In this paragraph, we:

1) Acknowledge the limitation related to single-manufacturer sourcing.
2) Emphasize that the main contribution is the generalizable LCA framework itself, which is independent of any specific brand.
3) Explicitly describe how healthcare institutions can practically use this framework to inform their procurement decisions by requesting data from various suppliers and running scenario analyses tailored to their local operations, thereby transforming the study from a static comparison into a dynamic decision-support tool.

We believe this addition powerfully articulates the strategic value and practical applicability of our research for promoting green procurement in healthcare.

**Comment 7:**

7. * Language and presentation

- The manuscript needs careful copyediting to remove repetition and awkward phrasing.

- Tighten the Abstract to remove duplicate statements of key findings.

- Define abbreviations on first use (CO2e, HLD, LTPS, functional unit).

- Make the Results section more concise; avoid restating exact table numbers in long prose.

**Response 7:**

We thank the reviewer for these valuable suggestions to improve the clarity and conciseness of our manuscript. We have undertaken a comprehensive copyediting process to address all points raised:

7.1. Comprehensive Language Editing: The entire manuscript has been carefully revised to eliminate repetition, awkward phrasing, and enhance overall fluency. We have focused on using precise academic language and streamlining sentence structures for better readability.

7.2. Tightened Abstract: The Abstract has been significantly condensed to remove duplicate statements of key findings. It now presents a more streamlined and impactful summary of the study.

7.3. Standardized Abbreviations: All abbreviations (CO₂e, HLD, LTPS, FU) have been explicitly defined upon their first use in both the abstract and the main text.

7.4. Concise Results Section: The Results section has been refined to avoid restating exact table and figure numbers in long prose. Findings are now stated directly, with references to supporting data presented succinctly in parentheses.

**Reviewer #4**

Interesting findings to support reduction in green gas effect from our daily use of medical equipment or devices and encourage green amongst medical practitioners. Reusable devices are expected to have lower carbon footprint than disposable devices (more solid waste). VL stylets is not widely available for every country hence it is difficult to understand the study when first read.

**Response :**

We sincerely thank the reviewer for raising this important point regarding the global applicability of the devices examined in our study. We fully acknowledge that the specific equipment analyzed—particularly the video stylet—may not yet be universally available across all healthcare settings worldwide.

We humbly wish to clarify that the three types of video intubation devices included in this study are widely adopted in China and represent the predominant categories of such devices currently available on the market. We recognize that this may limit the immediate generalizability of our findings in regions where these technologies are less common.

That said, we hope that the methodological framework developed in this work—based on a standardized life cycle assessment (LCA) approach incorporating functional unit definition, system boundary selection, and sensitivity analysis of key parameters such as device lifetime and sterilization efficiency—may still offer a transferable model for evaluating the environmental impact of other medical devices, even in different contexts.

We believe that as advanced medical technologies continue to diffuse globally, the analytical approach proposed here could serve as a useful reference for supporting sustainable procurement and operational decision-making in a broader range of settings.

We are deeply grateful for the reviewer’s insightful comments, which have helped us better contextualize the scope and contributions of this work.

We hope that these revisions address the reviewer’s concerns and enhance the completeness and academic value of the study.

Thank you once again for your valuable suggestions!
